# Phase 1 safety and pharmacodynamic study of lenalidomide combined with everolimus in patients with advanced solid malignancies with efficacy signal in adenoid cystic carcinoma

**DOI:** 10.1038/s41416-020-0988-2

**Published:** 2020-07-24

**Authors:** R. Donald Harvey, Bradley C. Carthon, Colleen Lewis, Mohammad S. Hossain, Chao Zhang, Zhengjia Chen, Wayne B. Harris, Olatunji B. Alese, Walid Shaib, Mehmet A. Bilen, David H. Lawson, Christina Wu, Conor E. Steuer, Bassel F. El-Rayes, Fadlo R. Khuri, Sagar Lonial, Edmund K. Waller, Suresh S. Ramalingam, Taofeek K. Owonikoko

**Affiliations:** 1 Department of Hematology and Medical Oncology, Emory University, Atlanta, GA USA; 2 Winship Cancer Institute of Emory University, Atlanta, GA USA; 3 Department of Statistics, Rollins School of Public Health, Emory University, Atlanta, GA USA; 4 American University of Beirut, Beirut, Lebanon

**Keywords:** Drug development, Targeted therapies

## Abstract

**Background:**

Purpose: The combination of a mammalian target of rapamycin inhibitor and lenalidomide showed enhanced preclinical cytotoxicity. We conducted a phase 1 study in advanced solid tumour patients to assess safety, efficacy and pharmacodynamic (PD) outcomes.

**Methods:**

We employed a 3+3 dose escalation design to establish the safety and recommended phase 2 doses (RP2D) of daily everolimus and lenalidomide in patients with advanced solid tumours. The starting doses were 5 and 10 mg, respectively, with planned escalation to maximum single-agent doses of 10 and 25 mg in the absence of dose-limiting toxicity. PD endpoints of lymphocyte subsets and immune cytokines were assessed in peripheral blood using multiparameter flow cytometry and LUMINEX assay. Efficacy was evaluated by cross-sectional imaging after every two cycles of treatment.

**Results:**

The study enrolled 44 patients, median age of 58 years and 28 males (63.6%). The RP2D was established as 10 and 25 mg daily continuously for everolimus and lenalidomide. Common (>5%) grade ≥3 adverse events included rash (19%), neutropenia (19%), hypokalaemia (11%) and fatigue (9%). Best efficacy outcomes in 36 evaluable patients were partial response in 5 (13.8%), stable disease in 24 (55.8%) and progressive disease in 7 (19.4%) patients. PD assessment revealed significant association of cytokine levels (interleukin-2 (IL2), IL21 and IL17), baseline activated and total CD8+ lymphocytes and change in B cell lymphocytes and activated NK cells with clinical benefit.

**Conclusions:**

The study demonstrated the safety of everolimus and lenalidomide with promising efficacy signal in thyroid and adenoid cystic cancers.

**Clinical Trial Registration:**

NCT01218555

## Background

The mammalian target of rapamycin (mTOR) signalling pathway is a clinically relevant target in many solid and haematologic malignancies. Preclinical studies have demonstrated that abrogation of mTOR signalling leads to tumour growth inhibition due to the central involvement of the mTOR pathway in myriad cellular processes, including cell growth, cell survival, protein synthesis and angiogenesis. Agents targeting the mTOR pathway such as temsirolimus and everolimus are standard treatments for advanced solid cancers. Combination strategies to improve the clinical efficacy of these agents have been explored both preclinically and in clinical trials.^1^ Improved efficacy was noted when everolimus was combined with biologically rational partners, such as anti-angiogenic agents, somatostatin analogues and anti-oestrogens.^2–4^

Lenalidomide is a second-generation immunomodulatory agent with improved tolerability and greater cytotoxicity than the original compound, thalidomide. Immune modulatory drugs, such as lenalidomide, inhibit ubiquitination of endogenous E3 ubiquitin ligase substrates by binding cereblon, a critical adaptor molecule required for substrate specificity of this ligase. Furthermore, the degradation of transcription factors Ikaros, Aiolos, casein kinase 1α and others contributes to the clinical activity of this class of agents.^5,6^ In addition to direct cytotoxicity resulting from the degradation of these transcription factors, lenalidomide also induces anti-angiogenic, epigenetic and inflammatory effects through its action on cancer cell–stromal interaction. Lenalidomide also induces cell cycle arrest and apoptosis, stimulates T cell-specific immune response and promotes expansion and enhanced cytotoxicity of natural killer (NK) cells.^7,8^ Historically, lenalidomide showed modest efficacy against solid tumours, such as refractory renal cell carcinoma (RCC), where it achieved a complete response (CR) in 3%, partial response (PR) in 8% and stable disease (SD) in 53% of patients when administered at the standard approved dose of 25 mg.^9^

In preclinical studies, cells exposed to mTOR inhibitors demonstrated increased expression of the phosphorylated AKT, which in turn activates downstream signalling through alternative pathways, including forkhead transcription factor and glycogen synthase kinase-3, that can bypass the mTOR complex.^10,11^ Immunomodulatory agents such as lenalidomide can overcome cellular tolerance of mTOR inhibition through putative mechanisms such as enhanced anti-tumour immunity and induction of E3 ligase. DEPTOR and other naturally occurring inhibitors of the mTOR signalling pathway are substrates of the E3 ubiquitin ligase, resulting in activation of this pathway.^12,13^ Targeting the E3 ligases through immunomodulatory agents is expected to result in accumulation of these inhibitors, and effective blockade of the mTOR pathway. The combination of lenalidomide and mTOR inhibitors was synergistic against several cancer cell lines in preclinical testing.^14,15^ We therefore tested the combination of lenalidomide and everolimus in patients with advanced solid malignancies. Specifically, we wanted to explore this combination in RCC due to known efficacy of mTOR inhibition and predicted additive and potentially synergistic activity with lenalidomide, as well as other tumour types where we might encounter promising efficacy signal.

## Methods

### Eligibility

Eligible adult patients were required to be ≥18 years of age, able and willing to provide informed consent and with histologic confirmation of a progressive solid malignancy. Patients were required to have at least one measurable site of disease according to the RECIST 1.1 criteria and must have discontinued anti-cancer therapy at least 4 weeks prior to initiation of treatment. Other eligibility criteria included Eastern Cooperative Oncology Group performance status of 0–2; adequate organ function indicated by: absolute neutrophil count ≥1500 cell/mm^3^, haemoglobin ≥9 g/dl, platelets ≥100,000 cells/mm^3^); serum creatinine within normal limits or creatinine clearance ≥60 ml/min/m^2^ if elevated; aspartate aminotransferase (AST) (SGOT) and alanine aminotransferase (ALT) (SGPT) <2 × upper limit of the normal range (ULN) or <5 × ULN in the presence of hepatic metastases. Salient exclusion criteria included inability to swallow pills; pregnancy or breast-feeding; prior exposure or known hypersensitivity to lenalidomide or everolimus; chronic treatment with systemic steroids or other immunosuppressive agent; and active therapy for HIV or infectious hepatitis type B or C. All study participants were registered into the mandatory Revlimid REMS^®^ program.

### Study design and treatment

We employed a standard 3 + 3 design to study escalating doses of daily, orally administered lenalidomide and everolimus. Dose escalation to the next cohort required 0 of 3 or ≤1 of 6 patients with dose-limiting toxicity (DLT). Five dose cohorts were planned starting at dose level one with lenalidomide 10 mg and everolimus 5 mg in a 28-day cycle up to maximum doses of 25 and 10 mg, respectively (Table 1). The maximum tolerated dose was the highest dose level of lenalidomide and everolimus at which <33% of the dose cohort experienced DLT after one cycle of therapy and was established as the recommended phase 2 dose (RP2D) for future studies. In the absence of intolerable toxicity, patients remained on treatment until objective tumour progression or withdrawal of consent. A planned expanded cohort of 15 patients with clear cell RCC, and subsequently, after an activity signal in the escalation phase, adenoid cystic carcinoma was filled to test for additional efficacy. Seven additional patients were able to be enrolled based on funding, for a total of 22 in expansion. The Emory University IRB approved the clinical trial protocol and all participants provided a written informed consent prior to undergoing any research-related procedures. The study was registered at www.clinicaltrials.gov with reference NCT01218555.

**Table 1:** Patient characteristics and clinical outcome.

Variable	Level	*N* (%) = 44
Age (years)	Median	58
Gender	Male	28 (63.6)
	Female	16 (36.4)
Race	White	32 (72.7)
	Asian or other	4 (9.1)
	African American	8 (18.2)
Ethnicity	Hispanic or Latino	3 (6.8)
	Non-Hispanic	40 (90.9)
	Unknown	1 (2.3)
DLT	No	18 (40.9)
	NE	3 (6.8)
	RP2D	22 (50.0)
	Yes	1 (2.3)
Histologic type	Adenoid cystic	15 (34.1)
	Renal cell carcinoma	9 (20.5)
	Thyroid cancer	5 (11.4)
	Other	15 (34.1)
Dose cohorts	1: Lenalidomide/everolimus (10/5 mg)	3 (7.0)
	2: Lenalidomide/everolimus (15/5 mg)	3 (7.0)
	3: Lenalidomide/everolimus (20/5 mg)	5 (12.8)
	4: Lenalidomide/everolimus (25/5 mg)	3 (7.0)
	5: Lenalidomide/everolimus (25/10 mg)	8 (18.6)
	RP2D: L (25 mg) and E (10 mg)	22 (51.2)
Best response	PD	7 (16.3)
	PR	5 (11.6)
	SD	24 (55.8)
	NE	7 (16.3)
OS (months)	Median	33 (1.22–73.59)
TTF (months)	Median	3.45 (0.46–35.49)
Number of cycles	Mean	7.77
	Median	5.00

Summary of demographic characteristics, tumour histology, dose cohort assignment and efficacy outcome in study patients.

*DLT* dose-limiting toxicity, *RP2D* recommended phase 2 dose, *SD* stable disease, *PR* partial response, *PD* pharmacodynamic, *NE* not evaluable, *TTF* time to treatment failure, *OS* overall survival.

### Adverse events and DLTs

Patients were evaluated and graded for treatment-emergent toxicities prior to starting a new cycle of treatment using the National Cancer Institute Common Terminology Criteria for Adverse Events (NCI CTCAE) Version 4.0. DLT was assessed in cycle 1 only and the following adverse events (AEs) were considered DLTs: grade 4 haematological toxicity lasting more than 7 days; grade 4 neutropenia of any duration in the presence of fever ≥38.5 °C; grade ≥3 nausea and/or vomiting despite standard supportive therapy; grade ≥3 non-haematologic toxicity excluding alopecia; inability to re-treat within 2 weeks of a scheduled cycle due to treatment-related AE; and inability to deliver all planned doses of lenalidomide and or everolimus during the first cycle due to an unexpected drug-related toxicity. Prophylactic growth factor support was not allowed during the DLT assessment period in the first cycle.

### Efficacy

All patients were required to have measurable disease by the RECIST criteria 1.1 and must have experienced objective disease progression on cross-sectional anatomic imaging obtained within 4 weeks of initiating treatment on study. Restaging scans were obtained after every two cycles during treatment. Efficacy was assessed and graded according to the RECIST 1.1 guidelines as CR, PR, SD or pharmacodynamic (PD). Overall survival (OS) was measured from the date of entry on study to date of death or censoring. Time to treatment failure (TTF) was defined as the time from treatment initiation to time of treatment discontinuation for any reason, including tumour progression or intolerable toxicity.

### Pharmacodynamic assessments

Peripheral blood samples collected from enrolled patients at baseline, the end of cycle 1 and at time of progression were employed for PD assessment using multicolour flow cytometry as previously described.^16^ Naive and activated TCRα/b+CD3+ T cell subsets were characterised by staining the red blood cell lysed whole blood with anti-human antibodies against CD3, CD4, CD8, CD69, ICOS, CD62L, CD69, and so on. The activation status of TCRα/b+CD3+ T cells were determined after 4 h in vitro stimulation with peripheral blood mononuclear cell at 37 °C with BD leucocytes activation cocktail in the presence of brefildin A following a standard staining protocol as previously described.^16^ The data were acquired in BD FACScanto and BD FACS Aria flow cytometers and analysed using the FlowJo software.

Circulating cytokines and chemokines (BDNF, NGF, ENA78, EOTAXIN, FGFB, GCSF, GMCSF, GROA, HGF, IFNB, IFNG, IL10, IL12P70, IL15, IL17A, IL17F, IL18, IL1B, IL1RA, IL2, IL21, IL22, IL23, IL27, IL31, IL4, IL5, IL6, IL7, IL8, IL9, IP10, LEPTIN, LIF, MCSF, MCP1, MCP3, MIG, MIP1A, MIP1B, PAI1, PDGFBB, RANTES, RESISTIN, CD40L, SCF, SDF1A, FASL, ICAM1, VCAM1, TGFA, TGFB, TNFA, TNFB, TRAIL, VEGF, VEGFD) were quantified in banked serum samples stored at −80 °C using the LUMINEX platform. This analysis was performed at the Immune Monitoring Centre at Stanford University as previously reported.^16^

### Statistics

Descriptive statistics were employed to summarise baseline subject characteristics, tumour types and adverse event experience. Subjects who received at least one dose of study drugs were included in the safety analysis. For numeric covariates, the mean and standard deviation were calculated and presented. Frequency and percentage were shown for categorical variables. Fisher’s exact test or chi-squared test was used to detect differences between categorical covariates, and analysis of variance test was used for numerical covariates. The univariate association of each covariate with OS or TTF was assessed using the Cox proportional hazards model. Kaplan–Meier plot and log-rank test also were presented for OS or TTF for relevant covariates. Statistical analysis was conducted using SAS Version 9.4.

## Results

### Patient characteristics and treatment delivery

The study enrolled a total of 44 subjects with a median age of 58 years (range 29–77 years). The majority of patients were male (63.6%) and of Caucasian ethnicity (72.7%). Twenty-two subjects were treated across five dose cohorts in the dose escalation phase of the study, while an additional cohort of 22 subjects with RCC and adenoid cystic carcinoma were treated at the RP2D in the dose expansion phase. Originally, the expansion cohort was designed to exclusively enrol patients with RCC; however, efficacy signal seen in adenoid cystic carcinoma led to a protocol amendment to include these patients as well. One patient treated in the dose escalation phase experienced a DLT, 18 patients tolerated treatment without DLT and 3 patients were not evaluable for DLT. The combination of lenalidomide and everolimus was well tolerated at the maximum doses tested (25 mg daily and 10 mg daily, respectively), which was declared the RP2D. Table 1 provides the details of patient demographics, tumour types, cohort and DLT experience on study.

### Safety and toxicity

The most commonly reported AEs (occurring in >10% of subjects) included fatigue (33%), rash (30%), anorexia (26%), diarrhoea (23%), nausea (21%), elevated AST (19%), cough (16%) and constipation (14%). Treatment-emergent grade 3 or 4 AEs included rash (19%), neutropenia (19%), hypokalaemia (11%), fatigue (9%), anaemia (5%), hypophosphataemia (5%) and thrombocytopenia (5%) (Table 2). There were no treatment-related deaths or death on study, but 18 of 44 patients had died as of the time of data analysis. The most common reason for discontinuation of study treatment was disease progression in 32 (72.7%) subjects, followed by adverse events in 9 (20.5%) subjects and patient’s decision in 3 (6.8%) subjects.

**Table 2:** Treatment-emergent adverse events by grade and frequency (*N* = 44).

Adverse events	Grade			
	1	2	3	4
ALT elevated	12%	9%		
Anaemia		14%	5%	
Anorexia	26%	7%		
AST elevated	19%			
Back pain		5%		
Cardiac troponin increased			2%	
Chest pain			2%	
Constipation	14%	9%		
Cough	16%			
Creatinine elevated		7%		
Dehydration			2%	
Diarrhoea	23%	12%	2%	
Dizziness			2%	
Dysgeusia	12%			
Dyspnoea			2%	
Erythema			2%	
Fatigue	33%	19%	9%	
Headache	12%			
Hypertriglyceridaemia	12%	7%		
Hypokalaemia		21%	9%	2%
Hypophosphataemia			5%	
Insomnia		5%		
Myocardial infarction			2%	
Nausea	21%	7%		
Neutropenia		9%	14%	5%
Pruritus	12%			
Rash	30%		19%	
Sinusitis	14%			
Sore throat	12%			
Thrombocytopenia		7%	5%	

Frequency of treatment-emergent adverse event regardless of causality graded according to NCI Common Terminology Criteria for Adverse Events (CTCAE) Version 4.

### Dose-limiting toxicities

One patient treated at the maximum tested dose (lenalidomide 25 mg and everolimus 10 mg) experienced a DLT of grade 4 myocardial infarction with troponin leak. This patient had a prior history of cardiac disease, thereby making attribution to investigational agents difficult. There was no protocol-defined DLT in the 22 additional patients treated at the RP2D.

### Anti-tumour efficacy

**Fig. 1:**
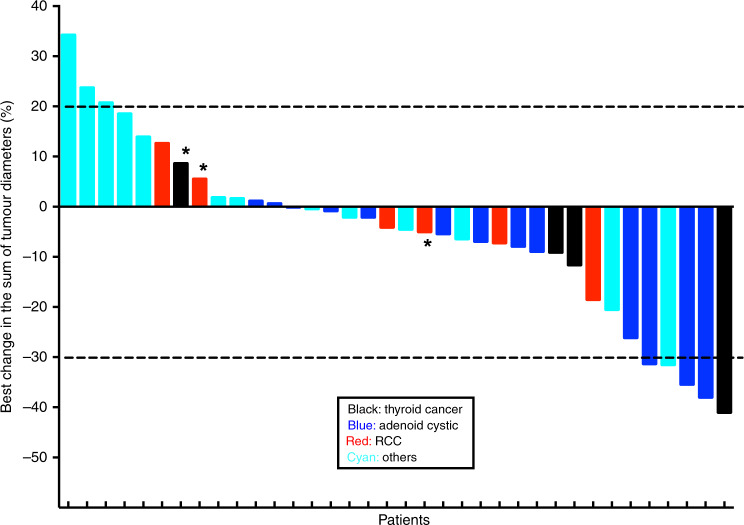
Waterfall plots for patients enrolled on study. There is objective tumour shrinkage in the majority of patient enrolled on study, although only five patients achieved partial response by RECIST 1.1 definition. Note that three patients without a 20% increase in the sum of target lesion diameters were classified as progressive disease based on the appearance of new lesions (identified by asterisks).

**Fig. 2:**
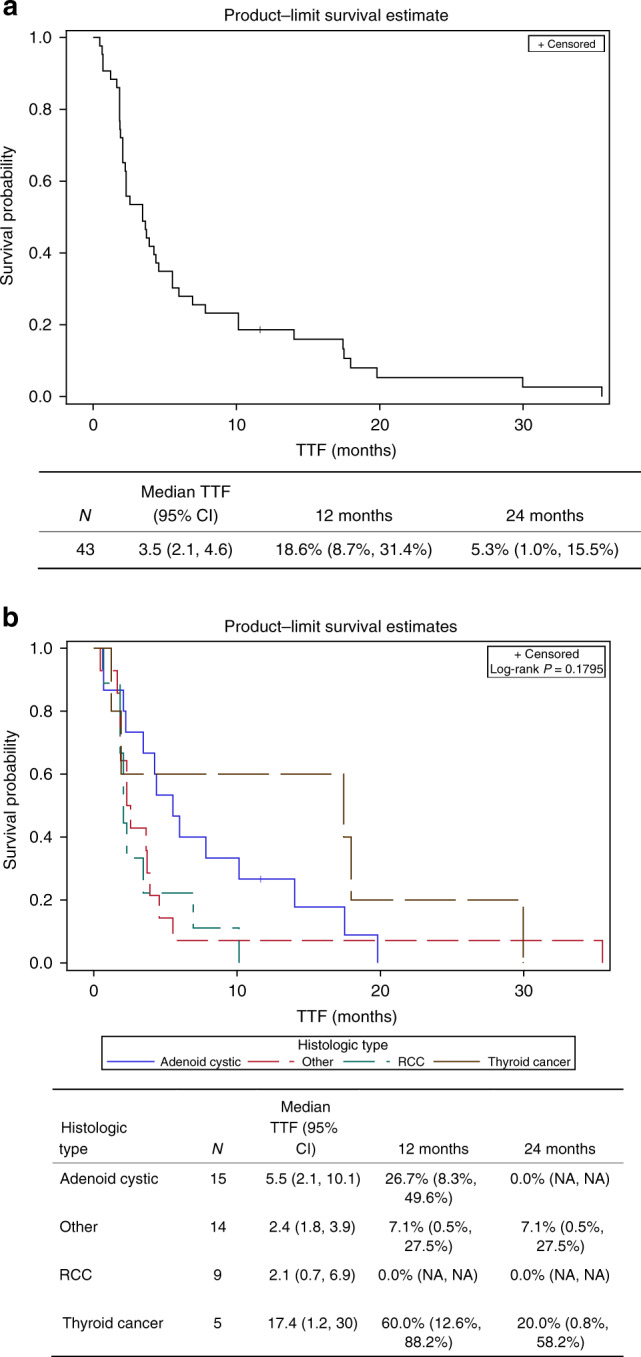
Overall efficacy and efficacy by tumor histology. **a** lKaplan–Meier time to event curve for time to treatment failure (TTF) for all patients enrolled on study. **b** Kaplan–Meier curves showing the time to treatment failure for patients by histologic category showing the best outcome for adenoid cystic and thyroid cancer patients.

**Fig. 3:**
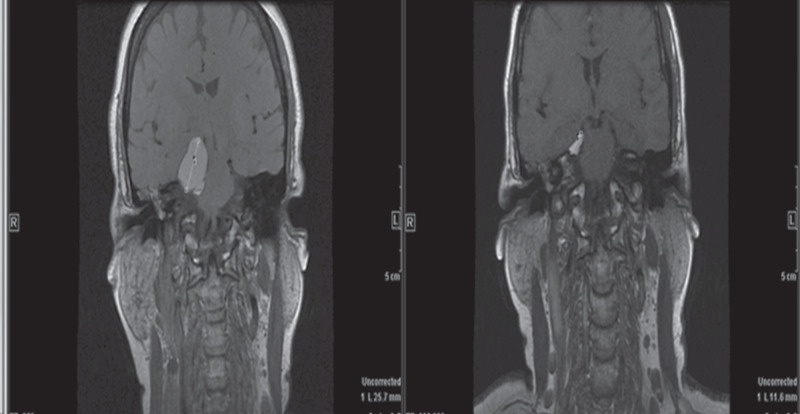
Intracranial response in a patient with adenoidcysitc carcinoma. Brain MRI in a patient with adenoid cystic salivary gland carcinoma showing significant shrinkage of intracranial tumour extension while receiving treatment with everolimus and lenalidomide.

One patient withdrew consent prior to meeting the requirement for efficacy assessment and was therefore excluded from efficacy analysis. The best response in 36 evaluable patients was PR in 5 (13.8%) patients (1 thyroid cancer, 1 salivary adenocarcinoma and 3 adenoid cystic carcinoma patients), SD in 24 (55.8%) patients and PD in 7 (19.4%) patients (Fig. 1). The median TTF was 3.5 (2.1, 4.6) months and ~18.6% of patients remained on treatment for at least 1 year (Fig. 2a). Patients with thyroid cancer and adenoid cystic carcinoma derived the greatest benefit from the combination with median TTF of 17.4 (1.2, 30) and 5.5 (2.1, 10.1) months, respectively, as compared to median TTF of 2.4 (1.8, 3.9) months for other tumour types (Fig. 2b). A patient with adenoid cystic carcinoma treated during the dose escalation phase of the study attained an impressive clinical and radiographic response in intracranial metastasis (Fig. 3).

### Survival

All patients, including those whose protocol therapy was discontinued, were followed for survival. The median OS was not reached (32.3 months, NA), while the estimated 1- and 2-year survival rates were 81.3% (66.1%, 90.2%) and 73.7% (57.5%, 84.5%), respectively.

### Correlative analysis

There was no significant difference in peripheral blood lymphocyte population subsets between pooled baseline and on-treatment samples. However, there was an association between TTF and specific lymphocyte subsets in ~34 patients with usable quality samples. Specifically, baseline activated CD8 (+) lymphocytes (*p* = 0.019); percentage of total lymphocytes that were CD8 (+) (*p* = 0.049); change in B cell lymphocytes (CD3 (−), CD19 (+); *p* < 0.001; and change in activated NK cells (CD16+, CD56+); *p* = 0.010 were significantly correlated with TTF (full datasets included in Supplementary Table S1).

Levels of serum cytokines were not significantly different between baseline and on-treatment samples. However, in 10 patients with matched baseline and on-treatment samples, comparison between patients who derived clinical benefit (PR, SD) and those who did not derive clinical benefit (PD) from the study treatment showed significantly different levels of modulation of IL2, IL17A, IL21, PDGFB and TRAIL in patients who attained clinical benefit compared to patients with PD (Table 3 and Supplementary Table S2).

**Table 3:** PD assessment of peripheral blood cytokines.

Serum cytokine	PD (*N* = 2) serum levels (ng/ml)	PR/SD (*N* = 8) serum levels (ng/ml)	*P* value
IL17A	176.38	−15.89	0.005
IL2	262.18	−2.95	0.006
IL21	1454.21	51.3	0.001
PDGFB	257.87	−42.7	0.033
TRAIL	1006.81	192.2	0.035

Changes between baseline and on-treatment levels of soluble immune mediators assessed by LUMINEX assay in patients with and without clinical benefit from the study treatment. Note that the majority of samples were excluded from analysis due to high coefficient of variation between replicate samples and or low level below the limit of quantitation of the LUMINEX assay.

## Discussion

mTOR inhibitors are established treatment options for cancers of the breast, kidney and pancreas.^2–4^ However, the efficacy recorded with these agents is generally modest, making it imperative to find additional strategies to further enhance the limited single-agent clinical efficacy. Based on preclinical data showing improved cytotoxicity of the combination of lenalidomide and mTOR inhibitors, we conducted this study to define the safety in patients with advanced solid tumours. We found the combination to be safe and well tolerated at the maximum doses in contrast to the experience in myeloma patients where the established RP2D was lower at 15 and 5 mg of lenalidomide and everolimus, respectively.^17^ Similarly, lenalidomide and temsirolimus required a dose reduction in temsirolimus to 15 mg at the RP2D.^18^ The successful combination at the highest single-agent dose of both agents in our study probably reflects differing patient characteristics, that is, solid tumour patients are more likely to have preserved marrow reserve in comparison to haematological malignancies.

The combination of everolimus and lenalidomide failed to show significant improvement over historical efficacy of everolimus in RCC, with a median TTF of 2.1 months. Surprisingly, however, there was signal of efficacy in adenoid cystic carcinoma with three objective responses and a median TTF of 5.5 months in comparison to historical experience with other targeted agents in this rare tumour.^19,20^ Similarly, a small cohort of five thyroid cancer patients achieved an impressive median TTF of 17.1 months, which is favourable against a median PFS of 10 months with single-agent everolimus in previous thyroid cancer trials.^21,22^ Our original intent was to study this combination in RCC as a strategy to improve on the modest efficacy of lenalidomide and everolimus as single agents. The surprising efficacy signal in a patient with adenoid cystic carcinoma during the dose escalation phase led to a protocol modification to allow enrolment of patients with adenoid cystic carcinoma into the expansion cohort.

Both everolimus and lenalidomide modulate immune effector cells, we therefore conducted PD analysis for changes in cellular and cytokine immune mediators in order to elucidate the potential role of immune modulation on anti-tumour efficacy of the regimen. As reported in previous studies of these agents, we did not observe any significant differences in the modulation of most lymphocyte subsets between baseline and on-treatment samples or by dose cohorts.^23^ We suspect that the long interval of 4 weeks between baseline and repeat samples could be partly responsible given the shorter time horizon of 1–2 weeks for immune cell modulation.^24^ Also, the inhibitory effect of everolimus on lymphocyte proliferation could have blunted any proliferative burst in lymphocyte and NK cells induced by lenalidomide.^25,26^ Nonetheless, when comparing patients who derived clinical benefit (SD or PR) to patients with best response of PD, there were notable differences in specific subsets of immune effector cells in peripheral blood [baseline activated CD8(+) T cells, change in B cells and activated NK cells]. The correlation noted between activated NK cell and clinical benefit replicated preclinical work that showed the critical role of activated NK cell in apoptosis induction by lenalidomide.^25^ Furthermore, there was significant modulation of interleukin family members (IL2, IL17A and IL21), TRAIL and PDGFB, and correlation with clinical benefit. While these observations are not conclusive, the modulation of tumour permissive IL17 along with other cytokines (IL2/IL21), which stimulate T cell proliferation, supports a significant contribution of immune-based mechanisms to the clinical efficacy of this regimen.^27–30^

In conclusion, the combination of everolimus and lenalidomide was safe and tolerable in patients with advanced solid malignancies. The regimen showed a promising signal of efficacy in adenoid cystic carcinoma and thyroid cancer patients and deserves further evaluation in larger studies.

## Supplementary information

Tables S1 and S2

## Data Availability

All relevant data pertaining to this study are included in the manuscript.
