# Supplementary material for: Phase 1 safety and pharmacodynamic study of lenalidomide combined with everolimus in patients with advanced solid malignancies with efficacy signal in adenoid cystic carcinoma
Source: Br J Cancer. 2020 Jul 24;123(8):1228–34. doi: 10.1038/s41416-020-0988-2 (PMC7553949; doi:10.1038/s41416-020-0988-2)
Supplement: Supplementary file 1 — Tables S1 and S2 [file 41416_2020_988_MOESM1_ESM.docx]

Legends:

**Table S1:**

Detailed results of lymphocyte subsets enumerated by flow cytometry in peripheral blood samples collected at baseline and on treatment including correlation with clinical efficacy and RECIST response categories

**Table S2:**

Detailed results of cytokines measured in peripheral blood samples collected at baseline and on treatment including correlation with clinical efficacy and RECIST response categories

| **Correlation of lymphocyte subsets (baseline counts and change from baseline versus on-treatment) with efficacy using median TTF as cut-point** | | | |
| --- | --- | --- | --- |
| **Lymphocyte subset** | **N** | **TTF**  **Hazard Ratio (95% CI)** | **P-value** |
| base T_% cd3+ | 34 | 1.00 (0.96-1.04) | 0.871 |
| base T_cd3+ | 34 | 1.00 (1.00-1.00) | 0.527 |
| base T_% cd4+ | 34 | 1.01 (0.97-1.05) | 0.543 |
| base T_cd4+ | 34 | 1.00 (1.00-1.00) | 0.382 |
| base T_% cd8+ | 34 | 0.99 (0.94-1.03) | 0.464 |
| base T_cd8+ | 34 | 1.00 (1.00-1.00) | 0.995 |
| base T_%cd69+cd4+ | 33 | 1.00 (0.95-1.05) | 0.871 |
| base T_cd69+,cd4+ | 34 | 1.00 (1.00-1.00) | 0.411 |
| base T_%Icos+cd4+ | 33 | 0.98 (0.84-1.14) | 0.750 |
| base T_Icos+,cd4+ | 34 | 1.00 (1.00-1.00) | 0.910 |
| base T_%cd69+cd8+ | 33 | 1.04 (0.95-1.14) | 0.391 |
| base T_cd69+,cd8+ | 34 | 1.00 (1.00-1.00) | 0.275 |
| base T_%Icos+cd8+ | 33 | 0.99 (0.72-1.34) | 0.924 |
| base T_Icos+cd8+ | 34 | 1.00 (1.00-1.00) | 0.837 |
| base B_% cd3- | 34 | 0.99 (0.95-1.04) | 0.720 |
| base B_% cd3-,cd19+ | 34 | 0.95 (0.87-1.04) | 0.279 |
| base B_cd3-,cd19+ | 34 | 1.00 (1.00-1.00) | 0.188 |
| base B_% Plasma | 34 | 1.03 (1.00-1.07) | 0.066 |
| base B_plasma | 34 | 1.00 (1.00-1.00) | 0.444 |
| base NK_% cd3+, cd94+ | 34 | 1.03 (0.99-1.07) | 0.108 |
| base NK_%cd3- | 34 | 0.99 (0.95-1.03) | 0.651 |
| base NK_%NK cd16+,cd56+ | 34 | 0.97 (0.90-1.05) | 0.508 |
| base NK | 35 | 1.00 (1.00-1.00) | 0.584 |
| base NK_%NK, cd94+ | 34 | 1.01 (1.00-1.03) | 0.134 |
| base NK,cd94+ | 35 | 1.00 (1.00-1.00) | 0.991 |
| base Treg_% cd3+ | 34 | 1.00 (0.97-1.04) | 0.872 |
| base Treg_%cd4+ | 34 | 1.01 (0.98-1.04) | 0.652 |
| base Treg_%cd4+,cd25+ | 34 | 1.00 (0.98-1.01) | 0.837 |
| base Treg_cd4+,cd25+ | 35 | 1.00 (1.00-1.00) | 0.823 |
| base Treg_% Treg | 34 | 1.00 (0.99-1.02) | 0.841 |
| base Treg | 35 | 1.00 (1.00-1.00) | 0.654 |
| base LA_% cd3+ | 32 | 1.02 (1.00-1.05) | 0.121 |
| base LA_% cd4+ | 32 | 1.00 (0.98-1.03) | 0.778 |
| base LA_% LA-IF+cd4+ | 32 | 0.97 (0.93-1.01) | 0.131 |
| base LA-IFN+,cd4+ | 32 | 1.00 (1.00-1.00) | 0.765 |
| base LA_% cd8+ | 31 | 0.98 (0.94-1.03) | 0.385 |
| **base LA_% LA-IF+cd8+** | **31** | **0.98 (0.96-1.00)** | **0.019** |
| base LA-IFN+,cd8+ | 31 | 1.00 (1.00-1.00) | 0.360 |
| base LA_% cd4+ | 32 | 1.00 (0.98-1.03) | 0.841 |
| base LA_% LA-GzB+cd4+ | 32 | 1.00 (0.99-1.01) | 0.900 |
| base LA-GzB+,cd4+ | 32 | 1.00 (1.00-1.00) | 0.429 |
| base LA_% cd8+ | 31 | 0.98 (0.93-1.02) | 0.279 |
| base LA_% LA-GzeB+cd8+ | 31 | 0.99 (0.98-1.01) | 0.349 |
| base LA-GzB+,cd4+ | 31 | 1.00 (1.00-1.00) | 0.784 |
| base GP_% cd3+ | 32 | 1.02 (0.99-1.06) | 0.216 |
| base GP_%cd3- | 32 | 0.98 (0.95-1.00) | 0.060 |
| base_GP__cd4 | 32 | 0.98 (0.95-1.00) | 0.060 |
| base GP_%GP-GzB+cd4+ | 32 | 0.99 (0.97-1.01) | 0.248 |
| base GP-GzB+,cd4+ | 32 | 1.00 (1.00-1.00) | 0.697 |
| **base GP_%cd8+** | **31** | **0.96 (0.92-1.00)** | **0.049** |
| base GP_%GP-GzB+cd8+ | 31 | 0.99 (0.98-1.00) | 0.168 |
| base GP-GzB+,cd8+ | 31 | 1.00 (1.00-1.00) | 0.571 |
| base GP_%cd3-,cd16+,cd56+ | 31 | 0.99 (0.95-1.03) | 0.652 |
| base GP_%GP-GzB+cd16+ | 31 | 0.99 (0.98-1.01) | 0.287 |
| base GP-GzB+,cd16+ | 34 | 1.00 (1.00-1.00) | 0.125 |
| base CD62%cd3+ | 34 | 1.00 (0.96-1.05) | 0.873 |
| base CD62cd3+ | 34 | 1.00 (1.00-1.00) | 0.523 |
| base CD62%cd4+ | 34 | 1.01 (0.97-1.05) | 0.565 |
| base CD62cd4+ | 34 | 1.00 (1.00-1.00) | 0.377 |
| base_CD62_cd8_ | 34 | 1.00 (1.00-1.00) | 0.377 |
| base CD62cd8+ | 34 | 1.00 (1.00-1.00) | 0.996 |
| base CD62%cd62L-cd4+ | 33 | 1.00 (0.98-1.02) | 0.802 |
| base CD62cd62L-,cd4+ | 33 | 1.00 (1.00-1.00) | 0.959 |
| base CD62%cd62L-cd8+ | 34 | 1.00 (0.99-1.02) | 0.707 |
| base CD62cd62L-,cd8+ | 34 | 1.00 (1.00-1.00) | 0.796 |
| change T_% cd3+ | 31 | 1.00 (0.97-1.03) | 0.929 |
| change_T_cd3 | 31 | 1.00 (0.97-1.03) | 0.929 |
| change T_% cd4+ | 31 | 0.99 (0.95-1.03) | 0.635 |
| change T_cd4+ | 31 | 1.00 (1.00-1.00) | 0.269 |
| change T_% cd8+ | 31 | 1.01 (0.97-1.05) | 0.768 |
| change T_cd8+ | 31 | 1.00 (1.00-1.00) | 0.186 |
| change T_%cd69+cd4+ | 33 | 1.03 (0.97-1.09) | 0.364 |
| change T_cd69+,cd4+ | 31 | 1.00 (1.00-1.00) | 0.547 |
| change T_%Icos+cd4+ | 31 | 0.90 (0.73-1.11) | 0.321 |
| change T_Icos+,cd4+ | 31 | 1.00 (1.00-1.00) | 0.545 |
| change T_%cd69+cd8+ | 31 | 1.06 (0.97-1.16) | 0.209 |
| change T_cd69+,cd8+ | 31 | 1.00 (1.00-1.00) | 0.198 |
| change T_%Icos+cd8+ | 31 | 0.71 (0.29-1.72) | 0.441 |
| change T_Icos+cd8+ | 31 | 1.00 (1.00-1.00) | 0.792 |
| change B_% cd3- | 31 | 1.01 (0.98-1.03) | 0.668 |
| change B_% cd3-,cd19+ | 31 | 1.03 (0.95-1.13) | 0.470 |
| **change B_cd3-,cd19+** | **32** | **1.00 (1.00-1.00)** | **<.001** |
| change B_% Plasma | 31 | 0.98 (0.95-1.02) | 0.298 |
| change B_plasma | 32 | 1.00 (1.00-1.00) | 0.587 |
| change NK_% cd3+, cd94+ | 31 | 0.98 (0.93-1.04) | 0.534 |
| change NK_%cd3- | 31 | 1.01 (0.99-1.03) | 0.493 |
| change NK_%NK cd16+,cd56+ | 31 | 1.00 (0.94-1.05) | 0.866 |
| change NK | 35 | 1.00 (1.00-1.00) | 0.339 |
| change NK_%NK, cd94+ | 31 | 1.00 (0.98-1.02) | 0.788 |
| change NK,cd94+ | 35 | 1.00 (1.00-1.00) | 0.102 |
| change Treg_% cd3+ | 31 | 1.01 (0.98-1.04) | 0.571 |
| change Treg_%cd4+ | 31 | 0.99 (0.95-1.04) | 0.790 |
| change Treg_%cd4+,cd25+ | 31 | 1.00 (0.98-1.01) | 0.760 |
| change Treg_cd4+,cd25+ | 33 | 1.00 (1.00-1.00) | 0.771 |
| change Treg_% Treg | 31 | 1.00 (0.98-1.01) | 0.560 |
| change Treg | 33 | 1.00 (1.00-1.00) | 0.908 |
| change LA_% cd3+ | 28 | 1.01 (0.98-1.05) | 0.409 |
| change LA_% cd4+ | 27 | 0.99 (0.96-1.03) | 0.618 |
| change LA_% LA-IF+cd4+ | 27 | 1.00 (0.97-1.03) | 0.877 |
| change LA-IFN+,cd4+ | 27 | 1.00 (1.00-1.00) | 0.922 |
| change LA_% cd8+ | 26 | 1.04 (0.99-1.09) | 0.121 |
| change LA_% LA-IF+cd8+ | 26 | 1.00 (0.99-1.02) | 0.806 |
| change LA-IFN+,cd8+ | 26 | 1.00 (1.00-1.00) | 0.200 |
| change LA_% cd4+ | 27 | 0.99 (0.96-1.03) | 0.595 |
| change LA_% LA-GzB+cd4+ | 27 | 1.00 (0.99-1.01) | 0.934 |
| change LA-GzB+,cd4+ | 28 | 1.00 (1.00-1.00) | 0.890 |
| change LA_% cd8+ | 26 | 1.05 (0.99-1.10) | 0.091 |
| change LA_% LA-GzeB+cd8+ | 26 | 1.01 (0.99-1.03) | 0.564 |
| change LA-GzB+,cd4+ | 27 | 1.00 (1.00-1.00) | 0.120 |
| change GP_% cd3+ | 28 | 1.02 (0.99-1.05) | 0.289 |
| change GP_%cd3- | 28 | 1.00 (0.97-1.02) | 0.723 |
| change GP_%cd4+ | 27 | 0.95 (0.91-1.00) | 0.056 |
| change GP_%GP-GzB+cd4+ | 27 | 1.00 (0.99-1.01) | 0.640 |
| change GP-GzB+,cd4+ | 27 | 1.00 (1.00-1.00) | 0.497 |
| change GP_%cd8+ | 26 | 1.05 (0.99-1.11) | 0.097 |
| change GP_%GP-GzB+cd8+ | 26 | 1.00 (0.99-1.02) | 0.429 |
| change GP-GzB+,cd8+ | 27 | 1.00 (1.00-1.00) | 0.197 |
| change GP_%cd3-,cd16+,cd56+ | 26 | 1.02 (0.97-1.07) | 0.544 |
| change GP_%GP-GzB+cd16+ | 26 | 1.00 (0.99-1.01) | 0.637 |
| **change GP-GzB+,cd16+** | **34** | **1.00 (1.00-1.00)** | **0.010** |
| change CD62%cd3+ | 31 | 1.00 (0.97-1.03) | 0.934 |
| change CD62cd3+ | 31 | 1.00 (1.00-1.00) | 0.167 |
| change CD62%cd4+ | 31 | 0.99 (0.94-1.04) | 0.644 |
| change CD62cd4+ | 31 | 1.00 (1.00-1.00) | 0.242 |
| change_CD62_cd8_ | 31 | 1.00 (1.00-1.00) | 0.242 |
| change_CD62cd8_ | 31 | 1.00 (1.00-1.00) | 0.242 |
| change CD62%cd62L-cd4+ | 31 | 0.99 (0.98-1.01) | 0.190 |
| change CD62cd62L-,cd4+ | 31 | 1.00 (1.00-1.00) | 0.715 |
| change CD62%cd62L-cd8+ | 32 | 1.00 (0.99-1.02) | 0.668 |
| change CD62cd62L-,cd8+ | 31 | 1.00 (1.00-1.00) | 0.224 |
| *Base = baseline value;*  *change = difference between baseline and on treatment samples*  *P<0.05 (without correction for multiple comparisons) considered clinically interesting* | | | |

| **Change in lymphocyte subsets using pooled unmatched samples in patients without (PD) and with (PR/SD) clinical benefit** | | | | |
| --- | --- | --- | --- | --- |
|  |  | **Response category** | |  |
| **Lymphocyte subset** | **Statistics** | **PD N=9** | **PR/SD N=28** | **Parametric P-value*** |
| change T_% cd3+ | N | 8 | 19 | 0.998 |
|  | Mean | 2.4 | 2.41 |  |
|  | Std Dev | 14.7 | 12.81 |  |
| change_T_cd3 | N | 8 | 19 | 0.998 |
|  | Mean | 2.4 | 2.41 |  |
|  | Std Dev | 14.7 | 12.81 |  |
| change T_% cd4+ | N | 8 | 19 | 0.513 |
|  | Mean | -1.86 | 0.47 |  |
|  | Std Dev | 4.36 | 9.45 |  |
| change T_cd4+ | N | 8 | 19 | 0.101 |
|  | Mean | 253813.7 | -136624.94 |  |
|  | Std Dev | 660393.12 | 491946.42 |  |
| change T_% cd8+ | N | 8 | 19 | 0.797 |
|  | Mean | 1.46 | 2.51 |  |
|  | Std Dev | 4.32 | 10.86 |  |
| change T_cd8+ | N | 8 | 19 | 0.339 |
|  | Mean | 105795.04 | -17528.08 |  |
|  | Std Dev | 412283.08 | 243252.54 |  |
| change T_%cd69+cd4+ | N | 8 | 21 | 0.895 |
|  | Mean | -0.94 | -1.28 |  |
|  | Std Dev | 6.24 | 6.18 |  |
| change T_cd69+,cd4+ | N | 8 | 19 | 0.446 |
|  | Mean | -83090.62 | -32162.68 |  |
|  | Std Dev | 252593.06 | 94744.58 |  |
| change T_%Icos+cd4+ | N | 8 | 19 | 0.161 |
|  | Mean | -1.09 | 0.04 |  |
|  | Std Dev | 1.5 | 1.98 |  |
| change T_Icos+,cd4+ | N | 8 | 19 | 0.404 |
|  | Mean | -13702.9 | -5106.98 |  |
|  | Std Dev | 26133.8 | 23186.59 |  |
| change T_%cd69+cd8+ | N | 8 | 19 | 0.703 |
|  | Mean | 0.67 | -0.04 |  |
|  | Std Dev | 3.15 | 4.76 |  |
| change T_cd69+,cd8+ | N | 8 | 19 | 0.992 |
|  | Mean | -14812.6 | -15208.81 |  |
|  | Std Dev | 125884.17 | 75029.6 |  |
| change T_%Icos+cd8+ | N | 8 | 19 | 0.183 |
|  | Mean | -0.24 | 0.11 |  |
|  | Std Dev | 0.49 | 0.64 |  |
| change T_Icos+cd8+ | N | 8 | 19 | 0.375 |
|  | Mean | -2594.6 | -362.11 |  |
|  | Std Dev | 5249.27 | 6087.67 |  |
| change B_% cd3- | N | 8 | 19 | 0.960 |
|  | Mean | 1.28 | 0.95 |  |
|  | Std Dev | 13.58 | 16.08 |  |
| **change B_% cd3-,cd19+** | **N** | **8** | **19** | **0.035** |
|  | Mean | 1.38 | -2 |  |
|  | Std Dev | 3.2 | 3.74 |  |
| **change B_cd3-,cd19+** | **N** | **8** | **20** | **0.001** |
|  | Mean | 114219.39 | -128352.04 |  |
|  | Std Dev | 229399.15 | 129403.2 |  |
| change B_% Plasma | N | 8 | 19 | 0.394 |
|  | Mean | -5.19 | 0.97 |  |
|  | Std Dev | 13.2 | 18.06 |  |
| change B_plasma | N | 8 | 20 | 0.661 |
|  | Mean | -2016.23 | -1285.52 |  |
|  | Std Dev | 6135.44 | 2722.92 |  |
| change NK_% cd3+, cd94+ | N | 8 | 19 | 0.940 |
|  | Mean | -1.12 | -0.88 |  |
|  | Std Dev | 5.66 | 8.17 |  |
| change NK_%cd3- | N | 8 | 19 | 0.342 |
|  | Mean | 2.9 | -4.54 |  |
|  | Std Dev | 19.75 | 17.59 |  |
| change NK_%NK cd16+,cd56+ | N | 8 | 19 | 0.742 |
|  | Mean | -0.66 | -1.52 |  |
|  | Std Dev | 3.19 | 6.92 |  |
| change NK | N | 9 | 22 | 0.155 |
|  | Mean | 41546.57 | -156192.14 |  |
|  | Std Dev | 154830.42 | 390359.78 |  |
| change NK_%NK, cd94+ | N | 8 | 19 | 0.113 |
|  | Mean | -12.67 | -0.16 |  |
|  | Std Dev | 17.26 | 18.41 |  |
| change NK,cd94+ | N | 9 | 22 | 0.149 |
|  | Mean | 18230.3 | -34259.69 |  |
|  | Std Dev | 101082.92 | 84686.19 |  |
| change Treg_% cd3+ | N | 8 | 19 | 0.739 |
|  | Mean | 3.57 | 1.85 |  |
|  | Std Dev | 16.03 | 10.17 |  |
| change Treg_%cd4+ | N | 8 | 19 | 0.729 |
|  | Mean | 1.25 | -0.01 |  |
|  | Std Dev | 6.24 | 9.24 |  |
| change Treg_%cd4+,cd25+ | N | 8 | 19 | 0.072 |
|  | Mean | -13.3 | 8.12 |  |
|  | Std Dev | 27.62 | 26.77 |  |
| change Treg_cd4+,cd25+ | N | 9 | 20 | 0.108 |
|  | Mean | -83024.44 | 35655.64 |  |
|  | Std Dev | 185454.04 | 174426.15 |  |
| change Treg_% Treg | N | 8 | 19 | 0.841 |
|  | Mean | 6.2 | 8.56 |  |
|  | Std Dev | 17.39 | 30.56 |  |
| change Treg | N | 9 | 20 | 0.255 |
|  | Mean | -6352.19 | 16075.59 |  |
|  | Std Dev | 24455.7 | 55032.06 |  |
| change LA_% cd3+ | N | 8 | 16 | 0.601 |
|  | Mean | 3.51 | 0.52 |  |
|  | Std Dev | 15.54 | 11.63 |  |
| change LA_% cd4+ | N | 8 | 15 | 0.181 |
|  | Mean | -1.36 | 6.81 |  |
|  | Std Dev | 9.61 | 15.05 |  |
| change LA_% LA-IF+cd4+ | N | 8 | 15 | 0.434 |
|  | Mean | -4.23 | 0.76 |  |
|  | Std Dev | 19.7 | 10.55 |  |
| change LA-IFN+,cd4+ | N | 8 | 15 | 0.589 |
|  | Mean | -565.69 | -41375.43 |  |
|  | Std Dev | 242751.45 | 117243.57 |  |
| change LA_% cd8+ | N | 8 | 15 | 0.106 |
|  | Mean | 5.86 | -1.71 |  |
|  | Median | 1.9 | -0.4 |  |
|  | Min | -8.4 | -22.9 |  |
|  | Max | 28.9 | 15.4 |  |
|  | Std Dev | 12.39 | 8.96 |  |
| change LA_% LA-IF+cd8+ | N | 8 | 15 | 0.969 |
|  | Mean | -0.22 | -0.73 |  |
|  | Std Dev | 30.74 | 29.64 |  |
| change LA-IFN+,cd8+ | N | 8 | 15 | 0.179 |
|  | Mean | 76756.3 | -27725.32 |  |
|  | Std Dev | 233599.49 | 129752.5 |  |
| change LA_% cd4+ | N | 8 | 15 | 0.264 |
|  | Mean | -1.09 | 5.68 |  |
|  | Std Dev | 9.78 | 14.99 |  |
| change LA_% LA-GzB+cd4+ | N | 8 | 15 | 0.337 |
|  | Mean | -0.89 | 13.48 |  |
|  | Std Dev | 22.72 | 37.61 |  |
| change LA-GzB+,cd4+ | N | 8 | 16 | 0.867 |
|  | Mean | 124207.46 | 91962.88 |  |
|  | Std Dev | 518746.01 | 397809.63 |  |
| change LA_% cd8+ | N | 8 | 15 | 0.171 |
|  | Mean | 5.67 | -0.36 |  |
|  | Std Dev | 10.82 | 9.13 |  |
| change LA_% LA-GzeB+cd8+ | N | 8 | 15 | 0.401 |
|  | Mean | -0.3 | 8.49 |  |
|  | Std Dev | 30 | 19.32 |  |
| change LA-GzB+,cd4+ | N | 8 | 16 | 0.376 |
|  | Mean | 102213.76 | 16655.1 |  |
|  | Std Dev | 304983.48 | 163793.13 |  |
| change GP_% cd3+ | N | 8 | 16 | 0.793 |
|  | Mean | 4.36 | 2.8 |  |
|  | Std Dev | 15.67 | 12.45 |  |
| change GP_%cd3- | N | 8 | 16 | 0.780 |
|  | Mean | -2.88 | -4.97 |  |
|  | Std Dev | 16.86 | 17.22 |  |
| change GP_%cd4+ | N | 8 | 15 | 0.690 |
|  | Mean | 0.48 | 2.26 |  |
|  | Std Dev | 12.17 | 8.86 |  |
| change GP_%GP-GzB+cd4+ | N | 8 | 15 | 0.278 |
|  | Mean | -3.62 | 13.3 |  |
|  | Std Dev | 18.06 | 40.51 |  |
| change GP-GzB+,cd4+ | N | 8 | 15 | 0.308 |
|  | Mean | -58995.62 | 113750.2 |  |
|  | Std Dev | 173716.4 | 445851.85 |  |
| change GP_%cd8+ | N | 8 | 15 | 0.300 |
|  | Mean | 1.64 | -2.46 |  |
|  | Std Dev | 5.7 | 10.01 |  |
| change GP_%GP-GzB+cd8+ | N | 8 | 15 | 0.270 |
|  | Mean | -4.97 | 10.75 |  |
|  | Std Dev | 24.54 | 34.7 |  |
| change GP-GzB+,cd8+ | N | 8 | 16 | 0.863 |
|  | Mean | 11135.52 | 34054.44 |  |
|  | Std Dev | 376638.56 | 262658.53 |  |
| change GP_%cd3-,cd16+,cd56+ | N | 8 | 15 | 0.730 |
|  | Mean | 0.99 | 2.21 |  |
|  | Std Dev | 8.19 | 7.78 |  |
| change GP_%GP-GzB+cd16+ | N | 8 | 15 | 0.222 |
|  | Mean | -0.71 | 20.92 |  |
|  | Std Dev | 47.26 | 34.53 |  |
| change GP-GzB+,cd16+ | N | 9 | 22 | 0.602 |
|  | Mean | 32245.1 | -71389.57 |  |
|  | Std Dev | 537732.43 | 479990.33 |  |
| change CD62%cd3+ | N | 8 | 19 | 0.971 |
|  | Mean | 2.82 | 2.61 |  |
|  | Std Dev | 14.75 | 12.36 |  |
| change CD62cd3+ | N | 8 | 19 | 0.088 |
|  | Mean | 438822.5 | -192835.58 |  |
|  | Std Dev | 1139990.4 | 695946.72 |  |
| change CD62%cd4+ | N | 8 | 19 | 0.594 |
|  | Mean | -1.28 | 0.58 |  |
|  | Std Dev | 4.47 | 9.22 |  |
| change CD62cd4+ | N | 8 | 19 | 0.077 |
|  | Mean | 286134.38 | -125372.71 |  |
|  | Std Dev | 662157.54 | 467408.49 |  |
| change_CD62_cd8_ | N | 8 | 19 | 0.077 |
|  | Mean | 286134.38 | -125372.71 |  |
|  | Std Dev | 662157.54 | 467408.49 |  |
| change_CD62cd8_ | N | 8 | 19 | 0.077 |
|  | Mean | 286134.38 | -125372.71 |  |
|  | Std Dev | 662157.54 | 467408.49 |  |
| **change CD62%cd62L-cd4+** | **N** | **8** | **19** | **0.022** |
|  | Mean | -15.01 | 11.14 |  |
|  | Std Dev | 26.25 | 24.91 |  |
| change CD62cd62L-,cd4+ | N | 8 | 19 | 0.177 |
|  | Mean | -69165.81 | 87311.12 |  |
|  | Std Dev | 339593.07 | 232756.66 |  |
| change CD62%cd62L-cd8+ | N | 9 | 19 | 0.618 |
|  | Mean | -4.6 | 0.12 |  |
|  | Std Dev | 30.9 | 18.68 |  |
| change CD62cd62L-,cd8+ | N | 8 | 19 | 0.121 |
|  | Mean | 132753.47 | -91284.21 |  |
|  | Std Dev | 348726.36 | 324229.94 |  |
| *  *Parametric p-value calculated by ANOVA;*  *p<0.05 (without correction for multiple comparisons) considered clinically interesting* | | | | |

| **Comparison of serum cytokine levels (ng/ml) in matched samples collected at baseline and on treatment** | | | | |  |
| --- | --- | --- | --- | --- | --- |
|  |  | **Sample collection time point** | |  |  |
| **Cytokine** |  | **Baseline**  **N=10** | **Treatment**  **N=10** | **P-value*** |  |
| BDNF | N | 6 | 6 | 0.962 | |
|  | Mean | 121.92 | 148.6 |  |  |
|  | Std Dev | 98.4 | 60.86 |  |  |
| NGF | N | 5 | 4 | 0.882 | |
|  | Mean | 188.69 | 440.43 |  |  |
|  | Std Dev | 144.38 | 308.63 |  |  |
| ENA78 | N | 5 | 6 | 0.341 | |
|  | Mean | 392.97 | 713.42 |  |  |
|  | Std Dev | 319.89 | 882.1 |  |  |
| EOTAXIN | N | 4 | 4 | 0.566 | |
|  | Mean | 31.35 | 45.5 |  |  |
|  | Std Dev | 5.72 | 18.85 |  |  |
| FGFB | N | 1 | 4 |  | |
|  | Mean | 134.54 | 348.5 |  |  |
|  | Std Dev | NA | 150.16 |  |  |
| GCSF | N | 10 | 10 | 0.150 | |
|  | Mean | 122.01 | 221.95 |  |  |
|  | Std Dev | 59.16 | 224.56 |  |  |
| GMCSF | N | 5 | 4 | 0.567 | |
|  | Mean | 54.79 | 80.49 |  |  |
|  | Std Dev | 35.21 | 59.17 |  |  |
| GROA | N | 2 | 4 |  | |
|  | Mean | 45.55 | 151.54 |  |  |
|  | Std Dev | 34.63 | 66.93 |  |  |
| HGF | N | 10 | 10 | 0.382 | |
|  | Mean | 95.6 | 119.64 |  |  |
|  | Std Dev | 47.14 | 88.99 |  |  |
| IFNB | N | 8 | 8 | 0.202 | |
|  | Mean | 1050.42 | 696.93 |  |  |
|  | Std Dev | 2171.05 | 1485.73 |  |  |
| IFNG | N | 2 | 3 |  | |
|  | Mean | 42.94 | 173.41 |  |  |
|  | Std Dev | 12.93 | 90.3 |  |  |
| IL10 | N | 3 | 4 | 0.809 | |
|  | Mean | 66.66 | 102.57 |  |  |
|  | Std Dev | 30.56 | 60.02 |  |  |
| IL12P70 | N | 1 | 3 |  | |
|  | Mean | 25.83 | 47.86 |  |  |
|  | Std Dev | NA | 33.82 |  |  |
| IL15 | N | 6 | 5 | 0.540 | |
|  | Mean | 99.26 | 277.06 |  |  |
|  | Std Dev | 120.97 | 206.67 |  |  |
| IL17A | N | 6 | 6 | 0.599 | |
|  | Mean | 57.24 | 80.46 |  |  |
|  | Std Dev | 41.23 | 69.59 |  |  |
| IL17F | N | 7 | 8 | 0.935 | |
|  | Mean | 110.31 | 97.99 |  |  |
|  | Std Dev | 106.39 | 131.6 |  |  |
| IL18 | N | 3 | 4 | 0.264 | |
|  | Mean | 78.84 | 186.58 |  |  |
|  | Std Dev | 77.64 | 90.18 |  |  |
| IL1B | N | 1 | 2 |  | |
|  | Mean | 22.35 | 48.02 |  |  |
|  | Std Dev | NA | 29.01 |  |  |
| IL1RA | N | 8 | 6 | 0.242 | |
|  | Mean | 600.73 | 1737.24 |  |  |
|  | Std Dev | 656.76 | 1733.91 |  |  |
| IL2 | N | 6 | 4 | 0.412 | |
|  | Mean | 56.04 | 129.28 |  |  |
|  | Std Dev | 39.14 | 115.77 |  |  |
| IL21 | N | 8 | 7 | 0.288 | |
|  | Mean | 254.44 | 513.5 |  |  |
|  | Std Dev | 300.16 | 830.45 |  |  |
| IL22 | N | 4 | 7 | 0.357 | |
|  | Mean | 932.2 | 681.71 |  |  |
|  | Std Dev | 813.73 | 210.62 |  |  |
| IL23 | N | 1 | 5 |  | |
|  | Mean | 322.72 | 617.31 |  |  |
|  | Std Dev | NA | 268.21 |  |  |
| IL27 | N | 4 | 4 | 0.982 | |
|  | Mean | 633.58 | 1644.15 |  |  |
|  | Std Dev | 462.27 | 1091.03 |  |  |
| IL31 | N | 3 | 4 | 0.783 | |
|  | Mean | 290.52 | 480.24 |  |  |
|  | Std Dev | 225.48 | 313.37 |  |  |
| IL4 | N | 8 | 7 | 0.256 | |
|  | Mean | 106.3 | 251.79 |  |  |
|  | Std Dev | 97.1 | 279.07 |  |  |
| IL5 | N | 4 | 4 | 0.620 | |
|  | Mean | 106.79 | 309.25 |  |  |
|  | Std Dev | 91.28 | 153.35 |  |  |
| IL6 | N | 4 | 4 | 0.667 | |
|  | Mean | 196.82 | 355.02 |  |  |
|  | Std Dev | 183.11 | 200.06 |  |  |
| IL7 | N | 1 | 2 |  | |
|  | Mean | 21.38 | 33.48 |  |  |
|  | Std Dev | NA | 7.64 |  |  |
| IL8 | N | 1 | 4 |  | |
|  | Mean | 22.24 | 92.26 |  |  |
|  | Std Dev | NA | 46.79 |  |  |
| IL9 | N | 4 | 4 | 0.722 | |
|  | Mean | 101.76 | 494.49 |  |  |
|  | Std Dev | 68.03 | 313.11 |  |  |
| IP10 | N | 7 | 4 | 0.302 | |
|  | Mean | 64.52 | 144.43 |  |  |
|  | Std Dev | 52.88 | 47.44 |  |  |
| LEPTIN | N | 10 | 10 | 0.063 | |
|  | Mean | 6905.19 | 4556.18 |  |  |
|  | Std Dev | 5048.94 | 5238.29 |  |  |
| LIF | N | 2 | 2 |  | |
|  | Mean | 94.71 | 100.2 |  |  |
|  | Std Dev | 98.73 | 76.72 |  |  |
| MCSF | N | 9 | 7 | 0.128 | |
|  | Mean | 101.14 | 210.28 |  |  |
|  | Std Dev | 60.28 | 164.8 |  |  |
| MCP1 | N | 2 | 5 |  | |
|  | Mean | 22.27 | 43.17 |  |  |
|  | Std Dev | 1.44 | 16.12 |  |  |
| MCP3 | N | 9 | 8 | 0.157 | |
|  | Mean | 132.36 | 346.77 |  |  |
|  | Std Dev | 110.42 | 365.67 |  |  |
| MIG | N | 10 | 10 | 0.534 | |
|  | Mean | 799.19 | 948.01 |  |  |
|  | Std Dev | 712.62 | 1304.91 |  |  |
| MIP1A | N | 5 | 4 | 0.162 | |
|  | Mean | 77.67 | 221.43 |  |  |
|  | Std Dev | 54.65 | 183.09 |  |  |
| MIP1B | N | 5 | 4 | 0.057 | |
|  | Mean | 37.6 | 105.85 |  |  |
|  | Std Dev | 15.55 | 51.45 |  |  |
| **PAI1** | **N** | **10** | **10** | **0.017** | |
|  | Mean | 6896.06 | 10848.55 |  |  |
|  | Std Dev | 2817.33 | 5537.82 |  |  |
| PDGFBB | N | 10 | 10 | 0.777 | |
|  | Mean | 143.1 | 160.51 |  |  |
|  | Std Dev | 137.47 | 190.26 |  |  |
| RANTES | N | 7 | 7 | 0.054 | |
|  | Mean | 30.25 | 45.11 |  |  |
|  | Std Dev | 5.95 | 15.82 |  |  |
| RESISTIN | N | 10 | 10 | 0.647 | |
|  | Mean | 2163.36 | 2383.26 |  |  |
|  | Std Dev | 953.62 | 976.01 |  |  |
| CD40L | N | 9 | 10 | 0.464 | |
|  | Mean | 303.46 | 244.57 |  |  |
|  | Std Dev | 530.25 | 434.95 |  |  |
| SCF | N | 4 | 4 | 0.915 | |
|  | Mean | 34.18 | 52.16 |  |  |
|  | Std Dev | 13.1 | 22.64 |  |  |
| SDF1A | N | 9 | 8 | 0.323 | |
|  | Mean | 1137.11 | 2203.03 |  |  |
|  | Std Dev | 1536.66 | 4025.81 |  |  |
| FASL | N | 1 | 4 |  | |
|  | Mean | 46.99 | 71.79 |  |  |
|  | Std Dev | NA | 40.39 |  |  |
| ICAM1 | N | 10 | 10 | 0.504 | |
|  | Mean | 4047.82 | 4852.19 |  |  |
|  | Std Dev | 3031.44 | 3929.93 |  |  |
| VCAM1 | N | 10 | 10 | 0.348 | |
|  | Mean | 2538531.58 | 2305487.33 |  |  |
|  | Std Dev | 864523.36 | 1071052.02 |  |  |
| TGFA | N | 3 | 3 |  | |
|  | Mean | 46.19 | 60.7 |  |  |
|  | Std Dev | 21.88 | 14.84 |  |  |
| TGFB | N | 4 | 4 | 0.916 | |
|  | Mean | 75.74 | 180.09 |  |  |
|  | Std Dev | 57.66 | 168.57 |  |  |
| TNFA | N | 10 | 10 | 0.344 | |
|  | Mean | 56.89 | 76.62 |  |  |
|  | Std Dev | 27.84 | 64.7 |  |  |
| TNFB | N | 2 | 4 |  | |
|  | Mean | 71.43 | 316.77 |  |  |
|  | Std Dev | 67.38 | 154.86 |  |  |
| TRAIL | N | 7 | 5 | 0.157 | |
|  | Mean | 100.07 | 472.46 |  |  |
|  | Std Dev | 90.24 | 367.17 |  |  |
| VEGF | N | 6 | 7 | 0.640 | |
|  | Mean | 407.96 | 537.56 |  |  |
|  | Std Dev | 712.54 | 393.74 |  |  |
| VEGFD | N | 7 | 8 | 0.583 | |
|  | Mean | 61.34 | 74.62 |  |  |
|  | Std Dev | 35.9 | 45.73 |  |  |

| **Comparison of change in serum cytokine levels (ng/ml) in matched samples collected at baseline and on treatment from patients with different response to treatment** | | | | |
| --- | --- | --- | --- | --- |
|  |  | **Response Category** | |  |
| **Covariate** | **Statistics** | **PD N=2** | **PR/SD N=8** | **P-value** |
| change_BDNF | N | 1 | 3 | 0.852 |
|  | Mean | 13.83 | -7.07 |  |
| change_ENA78 | N | 1 | 3 | 0.681 |
|  | Mean | 64.51 | 543.54 |  |
| change_GCSF | N | 2 | 8 | 0.637 |
|  | Mean | 164.98 | 83.68 |  |
| change_HGF | N | 2 | 8 | 0.079 |
|  | Mean | 115.14 | 1.26 |  |
| **change_IL17A** | **N** | **1** | **4** | **0.005** |
|  | Mean | 176.38 | -15.89 |  |
| change_IL17F | N | 2 | 5 | 0.382 |
|  | Mean | 47.65 | -23 |  |
| change_IL1RA | N | 2 | 4 | 0.335 |
|  | Mean | 2218.12 | 441.93 |  |
| **change_IL2** | **N** | **1** | **3** | **0.006** |
|  | Mean | 262.18 | -2.95 |  |
| **change_IL21** | **N** | **1** | **5** | **0.001** |
|  | Mean | 1454.21 | 51.3 |  |
| change_IL4 | N | 1 | 5 | 0.083 |
|  | Mean | 659.45 | 68.83 |  |
| change_IP10 | N | 1 | 3 | 0.383 |
|  | Mean | 169.13 | 34.2 |  |
| change_LEPTIN | N | 2 | 8 | 0.763 |
|  | Mean | -3079.41 | -2166.42 |  |
| change_MCSF | N | 1 | 6 | 0.171 |
|  | Mean | 299.89 | 67.67 |  |
| change_MCP3 | N | 2 | 6 | 0.665 |
|  | Mean | 307 | 165.57 |  |
| change_MIG | N | 2 | 8 | 0.138 |
|  | Mean | 843.24 | -24.79 |  |
| change_MIP1A | N | 1 | 3 | 0.053 |
|  | Mean | 339.34 | 65.19 |  |
| change_MIP1B | N | 1 | 2 | 0.247 |
|  | Mean | 114.19 | 60.16 |  |
| change_PAI1 | N | 2 | 8 | 0.848 |
|  | Mean | 3383.65 | 4094.7 |  |
| **change_PDGFBB** | **N** | **2** | **8** | **0.033** |
|  | Mean | 257.87 | -42.7 |  |
| change_RANTES | N | 2 | 4 | 0.550 |
|  | Mean | 24.31 | 14.03 |  |
| change_RESISTIN | N | 2 | 8 | 0.465 |
|  | Mean | 950.13 | 37.35 |  |
| change_CD40L | N | 2 | 7 | 0.147 |
|  | Mean | 94 | -73.05 |  |
| change_SDF1A | N | 2 | 6 | 0.140 |
|  | Mean | 3296.59 | 172.75 |  |
| change_ICAM1 | N | 2 | 8 | 0.724 |
|  | Mean | 1691.38 | 582.63 |  |
| change_TGFB | N | 1 | 2 | 0.429 |
|  | Mean | -94.88 | 59.22 |  |
| change_TNFA | N | 2 | 8 | 0.094 |
|  | Mean | 85.87 | 3.2 |  |
| **change_TRAIL** | **N** | **1** | **3** | **0.035** |
|  | Mean | 1006.81 | 192.2 |  |
| change_VEGF | N | 2 | 4 | 0.624 |
|  | Mean | -104.53 | 300.57 |  |
| change_VEGFD | N | 1 | 4 | 0.156 |
|  | Mean | -51.83 | 29.42 |  |
| *Change = change in cytokine level between baseline and on-treatment sample*  *P<0.05 (without correction for multiple comparisons) considered clinically interesting* | | | | |
